# The efficacy of aquatic therapy in stroke rehabilitation: A protocol for systematic review and meta-analysis

**DOI:** 10.1097/MD.0000000000027825

**Published:** 2021-12-03

**Authors:** Yumei Li, Gang Zheng

**Affiliations:** Department of Rehabilitation, The Affiliated People's Hospital of Ningbo University, Zhejiang, China, 315040.

**Keywords:** aquatic therapy, meta-analysis, prognosis, rehabilitation, stroke

## Abstract

**Background:**

Few studies have reported the clinical effect of aquatic therapy in stroke rehabilitation. Therefore, we performed a protocol for systematic review and meta-analysis to assess the effectiveness of aquatic therapy for individuals affected by strokes.

**Methods:**

This protocol of systematic review and meta-analysis has been drafted under the guidance of the preferred reporting items for systematic reviews and meta-analyses protocols. This study will use the Cochrane Library, Web of Science, PubMed, Embase, Allied and Complementary Medicine Database (AMED), China Biomedical Literature Database (CBM), China National Knowledge Infrastructure (CNKI), China Science and Technology Journal Database (VIP), Wanfang Database, and Ongoing Clinical Trials Database. Quality assessment of the included studies was evaluated using the Cochrane risk of bias assessment tool. We performed the meta-analysis by RevMan 5.4 software.

**Results:**

The results of this systematic review and meta-analysis will be published in a peer-reviewed journal.

**Conclusion:**

Aquatic therapy may be a valid means for the rehabilitation of people affected by stroke.

Open Science Framework registration number: https://doi.org/10.17605/OSF.IO/ZKE3Y10.17605/OSF.IO/8UDV9.

## 1 Introduction

Stroke, known as an acute cerebrovascular event closely related to brain tissue injury, is one of the leading causes of death and disability for adult men and women worldwide.^[1,2]^ According to World Health Organization reports, every year, ∼15 million people have a stroke,^[3]^ leading to a substantial economic and material burdens on the patients, families, and society.

Aquatic therapy plays an important role in the rehabilitation protocols for patients affected by neurological disease.^[4,5]^ The biological effects of water immersion involve essentially all the homeostatic systems of the human body.^[6]^ These effects are ascribable to the hydrodynamic principles. Each immersed body reacts to specific physical laws that influence its behavior in static and dynamic conditions. The intrinsic characteristics of water (hydrostatic pressure, buoyancy, viscosity, density, and temperature) and the dynamic characteristics (flow resistance and turbulent flow) act as facilitators: they permit a person immersed in water to practice balanced and coordinated movements.^[7]^

The hydrostatic pressure and viscosity of water provide proprioceptive and sensory feedback different from those experimented on land. Buoyancy is a force that provides support making it possible for patients to realize movements that cannot be done on land.^[8,9]^ The microgravity environment allows patients to actively take part in exercise because of the relief of the body weight. With the absence of a stationary position of the body in water, muscles are continuously activated to stabilize the body. This makes possible the acquisition of strength, flexibility, and balance. Viscosity slows movements, so, the response time to re-acquire a balanced state after postural perturbations is extended, thus reducing falls.^[10,11]^

Currently, few studies have reported the clinical effect of aquatic therapy in stroke rehabilitation. Therefore, we performed a protocol for systematic review and meta-analysis to assess the effectiveness of aquatic therapy for individuals affected by strokes.

## 2 Methods

### 2.1 Study registration

The protocol of this review was registered in OSF (OSF registration number: 10.17605/OSF.IO/8UDV9). It is reported to follow the statement guidelines of preferred reporting items for systematic reviews and meta-analyses protocol.^[12]^ Since this study is on the basis of published studies, ethical approval is not required.

### 2.2 Searching strategy

This study will use the Cochrane Library, Web of Science, PubMed, Embase, Allied and Complementary Medicine Database (AMED), China Biomedical Literature Database (CBM), China National Knowledge Infrastructure (CNKI), China Science and Technology Journal Database (VIP), Wanfang Database, and Ongoing Clinical Trials Database. There is no definite time limit for the retrieval literature, and the languages are limited to Chinese and English. Keywords used for the literature search are “aquatic therapy,” “aquatic treatment,” “aquatic exercise,” and “hydrotherapy” related to the keyword “stroke” through the Boolean operator “AND.”

### 2.3 Eligibility criteria

We included trials that met all the following criteria for analysis:

1. randomized controlled trials (RCTs);
2. the objects were definitely diagnosed with stroke, and their ages were over 18 years old;
3. the intervention group received aquatic therapy, while the control group received traditional rehabilitation exercise;
4. The trials tested the effects of aquatic therapy on: gait, balance, muscular function/strength of the lower limbs, independency in activities of daily living, proprioception, health related quality of life, physiological indicators, and cardiorespiratory fitness.

Studies were excluded if: they were not fully accessible, they were duplicated citations, and they possessed a poor quality score as per the stated criteria.

### 2.4 Data selection

First, two investigators used Endnote X9 software to conduct a preliminary assessment of the title and abstract of each document in the database based on the established criteria for inclusion in the study to select eligible studies. After a preliminary assessment, the full text of the selected literature was evaluated, and the uncontrolled study, no randomization, inconsistent evaluation criteria, and similar data were excluded. Finally, the final included studies were exchanged and checked by researchers. If the two researchers disagree on the results of a study or eventual inclusion, we will resolve it through discussion or consultation with a third person.

### 2.5 Data extraction

Before data collection, the study team built a data extraction sheet. Two authors separately collected relevant information from each eligible study. The data extraction table mainly includes the following contents: research title, first author, year of publication, sample size, duration of disease, intervention measures, outcome indicators, adverse events, and so on. If a study has unclear or inadequate information, we will attempt to contact the authors via email.

### 2.6 Risk of bias assessment

Two investigators will separately assess the risk of bias of the included studies using the Cochrane risk of bias assessment tool. The evaluation of each study mainly included the following seven aspects: random sequence generation, allocation hiding, blinding of participants and personnel, blinding of outcome assessment, incomplete outcome data, incomplete outcome data, selective outcome reporting, and other biases. Finally, the bias of the study will be rated on three levels: “low,” “high,” and “ambiguous.” These even domains will be separately appraised by two reviews, and discrepancies will be addressed by consulting a third reviewer.

### 2.7 Statistical analysis

In this study, we will apply RevMan 5.4 software for statistical analysis. The risk ratio and 95% confidence intervals (CIs) were collected for enumeration data, while the mean difference or standardized mean difference and 95% CIs were used to calculate continuous outcome data. Heterogeneity among trials will be identified by the *I*^2^ and Chi-squared test statistics. If the included studies have high heterogeneity (*I*^2^ > 50%), we will use a random-effects model for pooling data across studies. Otherwise, a fixed-effects model will be used. To evaluate publication bias, we will construct a funnel plot using Cochrane software if the number of included studies is sufficient (>10 studies). A symmetrical funnel plot indicates no possibility of publication bias, whereas an asymmetrical funnel plot indicates a high possibility of publication bias. If we identify publication bias through analysis of the funnel plot, we will discuss possible reasons such as small-study effects.

## 3 Discussion

Stroke incidence has increased in recent years and causes permanent disability.^[13,14]^ In an effort to address problems related to disability, many people use pharmacologic interventions or participate in multidisciplinary rehabilitation programs soon after having a stroke. However, despite intensive rehabilitation efforts, only ∼5% to 20% achieve complete functional recovery.^[15,16]^ Thus, there still exists an urgent need for new inpatient and outpatient rehabilitation and training strategies that match the specific needs of people after stroke.

Aquatic therapy is a common treatment modality used to address the complexity of patients with neurological disorders with the goal to achieve optimal functional independence.^[17]^ The physical properties of hydrodynamics, such as buoyancy, viscosity and thermodynamics, appear to benefit mobility in populations with disabilities. Although aquatic therapy continues to be widely utilized in neurorehabilitation, there is a lack of evidence on its effectiveness on mobility in adults with neurological disorders. This protocol can provide definite evidence regarding the efficacy of aquatic therapy for individuals affected by strokes.

## Author contributions

**Conceptualization:** Gang Zheng.

**Funding acquisition:** Yumei Li.

**Writing – original draft:** Yumei Li.

**Writing – review & editing:** Gang Zheng.
